# Characterization of the complete chloroplast genome of *Dendrobium findlayanum* Par. et Rchb. f. 1874 (Orchidaceae)

**DOI:** 10.1080/23802359.2022.2062264

**Published:** 2022-04-08

**Authors:** Cui-Ying Chen, Hui Tan, Rui Chen, Feng-Ping Zhang

**Affiliations:** College of Traditional Chinese Medicine, Yunnan University of Chinese Medicine, Kunming, China

**Keywords:** *Dendrobium findlayanum*, complete chloroplast genome, illumina sequencing, phylogeny, Orchidaceae

## Abstract

*Dendrobium findlayanum* Par. et Rchb. f. 1874 has the high ornamental and medicinal value. Here, we report the first complete chloroplast genome of *D*. *findlayanum*. The complete chloroplast genome of *D*. *findlayanum* is 153,713 bp in length with 120 genes, including 75 protein-coding genes, 37 tRNA genes, and 8 rRNA genes. The total content of GC of the whole genome is 37.46%. Phylogenetic analysis indicated that *D*. *findlayanum* was closely related to other species in *Dendrobium*, and this study provides new genetic resources for species identification and phylogenetic analyses in *Dendrobium*.

The genus *Dendrobium* Sw. 1799 is one of the largest in the family Orchidaceae, it contains more than 1500 species, and mainly distributed in tropical and subtropical Asia and eastern Australia (Teixeira da Silva et al. 2016). A subset of *Dendrobium* species were widely used in in Chinese traditional medicine and global horticultural trade. The wild populations of *Dendrobium* species were severely threatened because of the over-exploitation, climate change and its own biological characteristics. Therefore, it was classified as one of the endangered taxa of China. *Dendrobium findlayanum* Par. et Rchb. f. 1874 has high ornamental values, which found in Yunnan Province, Southwest China. *D*. *findlayanum* was listed in the category of the Convention on International Trade in Endangered Species of Wild Fauna and Flora (CITES).

The complete chloroplast genome sequence is considered as useful genetic information for evolutionary studies for its conservation. Here we report the complete chloroplast genome of *D*. *findlayanum* in order to provide helpful molecular data on phylogenetic relationships and conservation of *Dendrobium*.

The leaf samples of *D*. *findlayanum* were obtained in the Kunming Institute of Botany, Chinese Academy of Sciences (Kunming, Yunnan, China; 25° 10′ N, 102° 41′E). Voucher specimens were deposited in the Herbarium of Kunming Institute of Botany, Chinese Academy of Sciences (Dr. Zhang, zhangfengping8008@163.com) (voucher number: 1519997). Research on the plant and the collection of plant material has been carried out in accordance with guidelines provided by the authors’ institution(s) and national or international regulations. Field studies have complied with local legislation and appropriate permissions/license were granted while taking samples from a preserved/protected land. Authors comply with the International Union for Conservation of Nature (IUCN) policies research involving species at risk of extinction (see Guidelines for appropriate uses of IUCN Red list data), the Convention on Biological Diversity and the Convention on the Trade in Endangered Species of Wild Fauna and Flora. The sample’s total genomic DNA was extracted from fresh leaves of *D*. *findlayanum* by using a modified CTAB method (Doyle and Doyle 1987), then the sequencing library was produced with Illumina NEBNext Ultra™ DNA Library Prep Kit (Illumina, San Diego, USA) and was sequenced on Illumina Novaseq 6000 platform. The raw data were used to assemble the complete chloroplast genome by SPAdes v.3.13.0 software (Bankevich et al. 2012). Finally, the assembled complete chloroplast genome was annotated via CpGAVAS2 (Shi et al. 2019). The new annotated complete chloroplast genome sequence has been submitted to GenBank (accession number: MZ424316).

The total length of genome of *D*. *findlayanum* is 153,713 bp, it contains a large single-copy (LSC) region of 86,815 bp and a small single-copy (SSC) region of 14,604 bp, which is linked by two inverted repeat regions (IRa and IRb) of 26,398 bp. The overall GC content is 37.46%, while GC of content of SSC, LSC, and IR regions is 33.39%, 35.09%, and 43.34%, respectively. There are 75 protein coding genes (PCGs), 37 tRNA genes and 8 rRNA genes.

To investigate the phylogenetic position of *Dendrobium findlayanum*, the complete chloroplast genome sequences of *D*. *findlayanum*, other 17 *Dendrobium* species and two *Pleione* species (outgroups) were aligned using MAFFT v7.450 software (Katoh and Standley 2013). Our results indicated that *D*. *findlayanum* was closely related to other species in *Dendrobium* (Figure 1). This newly reported chloroplast genome provides a good resource for molecular studies of *D*. *findlayanum*.

**Figure 1.**
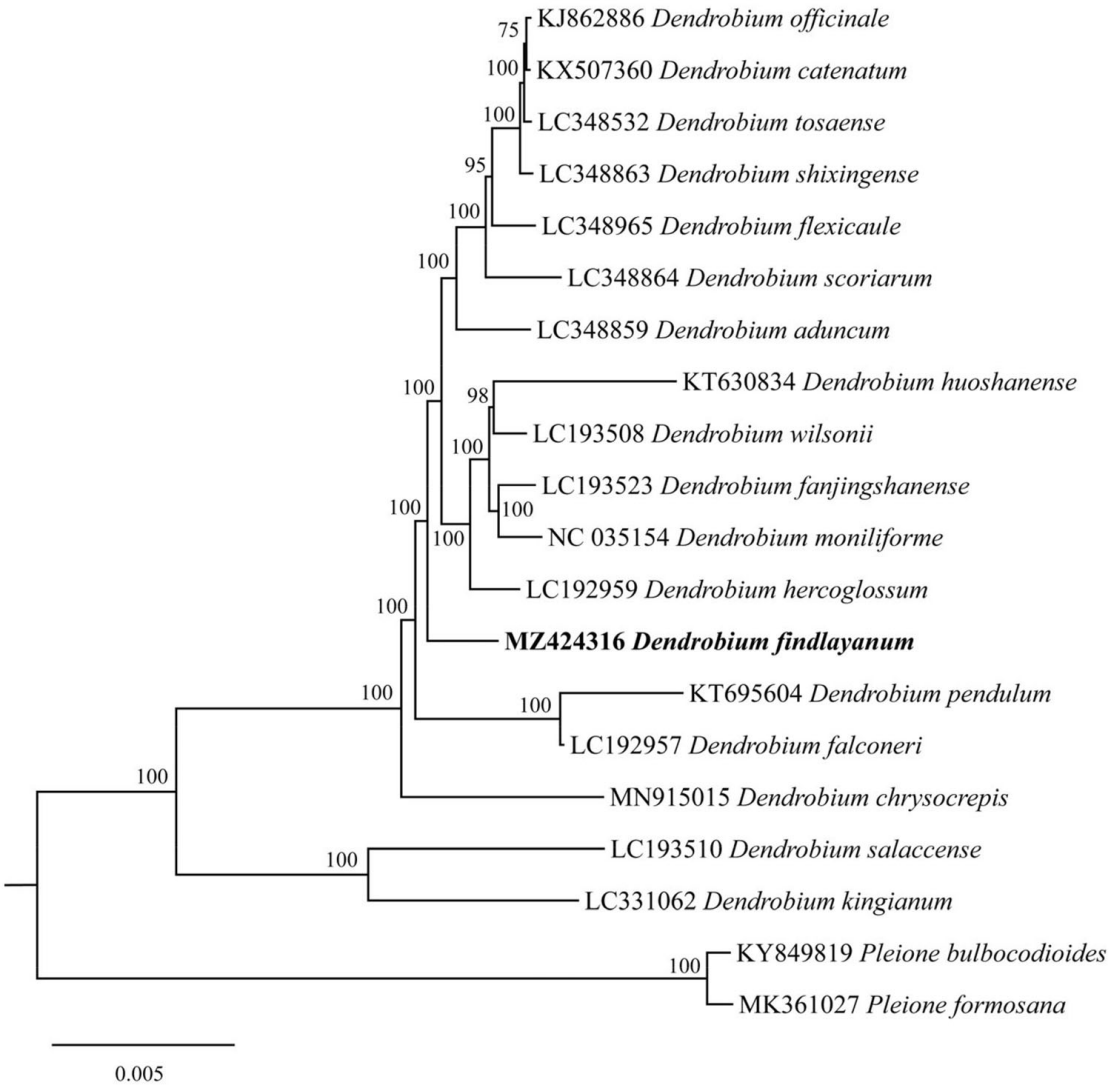
The phylogenetic tree was constructed by IQ-tree (v2.1.3, http://www.iqtree.org/) with the maximum-likelihood (ML) method based on the complete chloroplast genome sequences. The bootstrap values were based on 5000 ultrafast bootstraps and are shown next to the nodes. The position of *D. findlayanum* is shown in bold and black type.

## Data Availability

The genome sequence data that support the findings of this study are openly available in GenBank of NCBI at https://www.ncbi.nlm.nih.gov under the accession no. MZ424316. The associated BioProject, SRA, and Bio-Sample numbers are PRJNA771424, SAMN22310685 and SRR16350288, respectively.
